# Bacterial Ice Crystal Controlling Proteins

**DOI:** 10.1155/2014/976895

**Published:** 2014-01-20

**Authors:** Janet S. H. Lorv, David R. Rose, Bernard R. Glick

**Affiliations:** Department of Biology, University of Waterloo, Waterloo, ON, Canada N2L 3G1

## Abstract

Across the world, many ice active bacteria utilize ice crystal controlling proteins for aid in freezing tolerance at subzero temperatures. Ice crystal controlling proteins include both antifreeze and ice nucleation proteins. Antifreeze proteins minimize freezing damage by inhibiting growth of large ice crystals, while ice nucleation proteins induce formation of embryonic ice crystals. Although both protein classes have differing functions, these proteins use the same ice binding mechanisms. Rather than direct binding, it is probable that these protein classes create an ice surface prior to ice crystal surface adsorption. Function is differentiated by molecular size of the protein. This paper reviews the similar and different aspects of bacterial antifreeze and ice nucleation proteins, the role of these proteins in freezing tolerance, prevalence of these proteins in psychrophiles, and current mechanisms of protein-ice interactions.

## 1. Introduction

Throughout the planet, environmental temperatures can reach low to freezing levels. Organisms indigenous to these habitats are presented with potential desiccation, which can lead to potentially detrimental challenges such as decreased enzymatic rates, freezing, and aggregation of endogenous proteins [1, 2]. Besides hindering cellular processes, subzero temperatures induce ice formation, which can lead to cell death [3]. In some cases, intracellular ice crystals can rupture cells either physically or through osmotic pressure changes [4].

The temperature at which water freezes varies based on solution homogeneity [1]. Pure water was reported to freeze at −40°C. On the other hand, a heterogeneous water solution can contain additional molecules, such as dust particles and ice active bacteria, that act as seeds for ice nucleation [1, 5]. In these situations, a solution can freeze at high subzero temperatures, up to −2°C.

Cellular cryodamage incurred from freezing is dependent on freezing rate and ice crystal location [1]. For intracellular ice, a flash freezing rate (e.g., −100°C/min) minimizes potential damage while a slow rate is more detrimental [3]. Furthermore, with a slow rate of freezing, the internal ice acts as a solute drawing water into cells until they rupture. On the other hand, extracellular ice can cause membrane fracturing or shifting in osmotic pressures [6]. During external freezing, water solidification into ice removes available liquid water and concentrates extracellular solutes. This change simulates a high saline environment, drawing out internal water that is needed for cellular processes. Whether cellular damage is a result of extracellular ice formation or elevated salt concentration remains unclear [3].

One line of defense against cold temperature stress is the production of small molecules that can act as cryoprotectants [1, 7]. Common cryoprotectants include alcohol sugars, sugars, and amines. Secreted cryoprotectants can depress the freezing temperature of water in a colligative manner [3]. Intracellular cryoprotectants can regulate osmotic pressures by elevating internal solute concentrations, thus maintaining the osmolarity prior to freezing. Some cryoprotectants, such as albumin, can prevent cold denaturation of freeze labile proteins by decreasing the association between target enzymes and water molecules during freezing [1]. This cryoprotection is achieved via surrounding the target proteins. Other cryoprotective proteins such as cold shock proteins can assist in folding of essential proteins while preventing decreases in the expression of housekeeping genes.

An alternate strategy of cryoprotection is the production of antifreeze and ice nucleation proteins [1, 8]. Both classes of proteins are involved in ice crystal development by very similar mechanisms. In fact, some researchers argue that both proteins use the same mechanism, differing in function due to molecular size and concentration [9–13]. Not surprisingly, some bacteria have been found with both antifreeze and ice nucleation activity [4, 7, 14, 15]. Therefore, Kawahara [1] has proposed the term ice crystal controlling protein (ICC) to encompass both protein classes with an affinity for ice.

**Figure 1:**
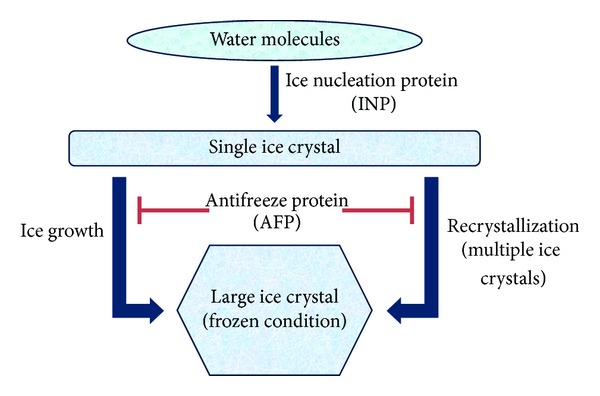
The interactions of ice crystal controlling proteins on the two stages of ice crystal growth. Ice nucleation proteins induce growth of single ice crystal nuclei, while antifreeze proteins inhibit further ice crystal growth. Blue lines represent ice growth direction at low subzero temperatures; red lines indicate inhibitory action.

Ice crystal formation occurs in two stages: ice nucleation and ice growth as shown in Figure 1 [12]. Each class of ice crystal controlling protein targets one of these two stages. Ice nucleation proteins (INPs) trigger ice crystal nucleation events at high subzero temperatures [1]. These events lead to formation of embryonic ice crystals and subsequent freezing. However, ice nucleation can be inhibited; some known anti-nucleating bacteria belong to the genus *Acinetobacter* or *Bacillus*. On the other hand, antifreeze proteins (AFPs) are a class of proteins characterized by the ability to bind to embryonic ice crystal and inhibit further growth [16]. Also, antifreeze proteins are known ice nucleation inhibitors, preventing the association of water with ice nucleators, subsequently preventing formation of embryonic ice crystals [12]. Therefore, antifreeze proteins maintain ice crystals at a manageable size.

Cold adaptations are not limited to cryoprotectants and ice crystal controlling proteins. A bacterium, *Paenibacillus *sp. C8, was found with ice binding capabilities but lacked detectable ice nucleation, thermal hysteresis, or ice recrystallization inhibition activity associated with ice crystal controlling proteins [15]. Also, *Bacilli *are known to survive harsh winters through sporulation. Therefore, bacteria may utilize more than one strategy to manage cold stress [6, 7].

### 1.1. Ice Crystals

**Figure 2:**
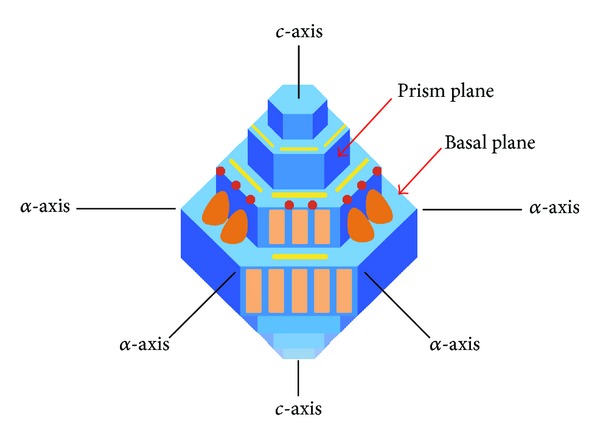
Potential interactions of antifreeze proteins on ice crystal surfaces. Antifreeze proteins (non-blue objects) bind to two major planes: prism and basal plane. Location of binding is dependent on the protein characteristics. Antifreeze proteins bound to the prism plane inhibit ice growth along the *α*-axes, but when bound to the basal plane *c*-axis ice growth is inhibited. Adapted from Kawahara [1].

During solidification into ice, water molecules are ordered into three-dimensional structures stabilized by an extensive network of hydrogen bonding. Because of the asymmetry of water molecules, spacing of oxygen atoms varies at different surfaces of this packed structure. These variable surfaces are considered ice planes and are the site of ice interaction [8]. There are two main ice crystal planes: the primary prism plane and basal plane (Figure 2). The primary prism plane lies perpendicular to the *α*-axes and parallel to the *c*-axis [16]. In this plane, oxygen atoms are spaced in regions 7.35 by 4.5 Ångstroms. Conversely, the basal plane lies perpendicular to the *c*-axis and parallel to the *α*-axes. Oxygen atoms of the basal planes are spaced in regions 7.8 by 4.5 Ångstroms. Typically, when ice grows, water molecules are ordered onto these planes creating another layer resulting in perpendicular growth. Consequently, when bound by ice crystal controlling proteins, ice growth can be altered [1, 17]. However, protein binding is not limited to the aforementioned planes and can bind to unspecified planes [18, 19].

### 1.2. Antifreeze Proteins

Antifreeze proteins are characterized by their ability to control the size and shape of ice crystals. Typically secreted, these proteins control extracellular ice by inhibiting ice growth [7]. Hypothesized to use an adsorption-inhibition mechanism, antifreeze proteins have two main activities: thermal hysteresis and ice recrystallization inhibition activity [1].

#### 1.2.1. Thermal Hysteresis

**Figure 3:**
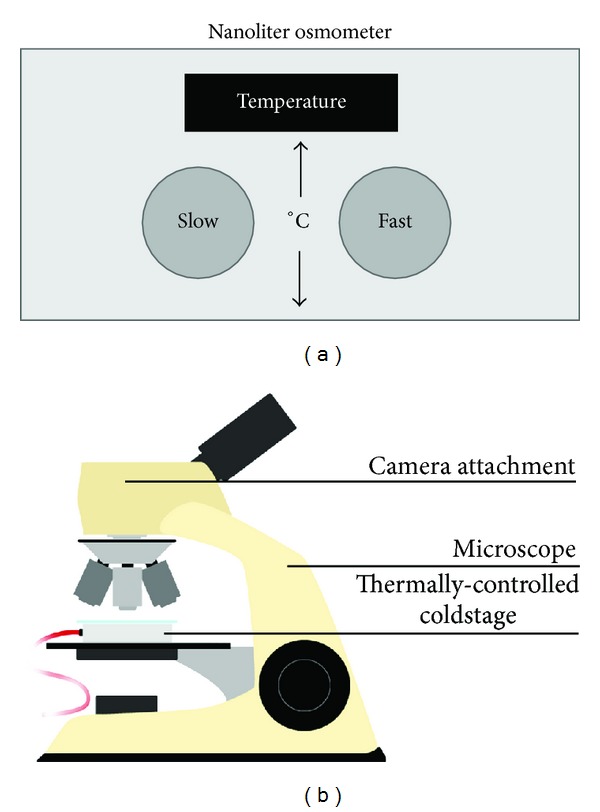
Instruments used to determine thermal hysteresis. The operator uses the nanoliter osmometer to carefully control the temperature of the cold plate: knobs are used to sensitively increase and decrease temperatures. A solution droplet placed in immersion oil is observed for ice crystals. Following flash freezing, the solution is melted until a single ice crystal remains to visually determine the melting and freezing temperature.

**Figure 4:**
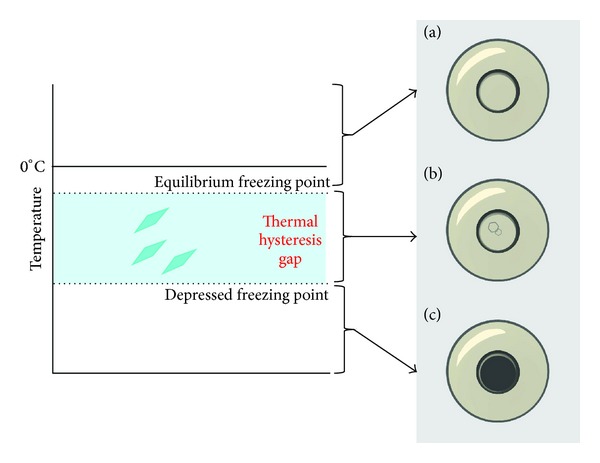
Antifreeze proteins depress the equilibrium freeze point, separating the freeze point from the melting point, creating a thermal hysteresis gap. In this gap, continual ice growth is inhibited and preventing freezing of the organism. Beyond this gap, freezing is inevitable. This can be observed with the nanoliter osmometer (a)–(c). (a) Above the equilibrium freeze/melting point ice crystals are absent. (b) After isolating individual ice crystals, within the gap ice crystals are seen to neither grow nor melt. (c) Beyond the gap, explosive growth of numerous ice crystals increases the opacity of the droplet, blocking microscope light.

Thermal hysteresis activity is the non-colligative depression of the freezing temperature, known as freezing hysteresis while also slightly elevating melting temperature, known as melting hysteresis [20]. When combined, a thermal hysteresis gap is formed between the two temperatures and is measured by a nanoliter osmometer (Figure 3). When ambient temperature falls within this gap, liquid water and ice crystals enter a supercooled state where ice crystals neither melt nor grow [21]. However, if the ambient temperature reaches below the depressed freezing temperature, the endpoint of the gap, ice growth resumes at an uncontrollable rate (Figure 4) [22]. Characterized by distinctive burst patterns, this is known as explosive ice growth and is physically detrimental to cells. Furthermore, explosive growth correlates to thermal hysteresis activity becoming increasingly more violent with greater thermal hysteresis gaps.

On the other hand, melting hysteresis can prevent ice crystal melting up to 0.18°C above the equilibrium melting temperature and is unrelated to freezing hysteresis [20]. This leads to superheated ice crystals. Similar to explosive growth, beyond the melting endpoint, ice crystals are rapidly melted. Its effect on organism survival has yet to be determined. As melting hysteresis is a recently discovered parameter, all reported thermal hysteresis activity in this review refers to freezing hysteresis.

Thermal hysteresis values vary widely between organisms [4]. Due to the non-colligative property of thermal hysteresis activity, comparisons are difficult. For example, insect antifreeze proteins have 10- to 20-fold greater activity than fish equivalents at similar concentrations [23]. Furthermore, plant and bacterial antifreeze proteins have been found to be consistently lower than insect or fish equivalents. Due to either the hyperbolic or sigmoidal trend of this activity, maximal thermal hysteresis values are usually reported. Typically, thermal hysteresis values range from 0.4 to 2.0°C in fish, 3 to 6°C in insects, 0.15 to 0.7°C in plants, and 0.1 to 0.3°C in bacteria [4, 24–27].

#### 1.2.2. Moderate Activity versus Hyperactivity

Initially discovered in insect antifreeze proteins, hyperactive antifreeze proteins are characterized by a large thermal hysteresis gap, melting hysteresis, primary prism plane and basal ice crystal plane binding, and a distinctive explosive burst pattern [20, 21, 28, 29]. Recently, fish and bacterial hyperactive antifreeze proteins have been discovered.

Thermal hysteresis gaps of hyperactive proteins are defined as significantly exceeding the maximal value of canonical fish antifreeze proteins [21]. Such differences are attributed to antifreeze protein coverage of the ice crystal. Moderate antifreeze proteins bind to the primary prism plane leaving the basal plane uncovered [30]. This lack of coverage permits the ice crystal to grow parallel to the *c*-axis. On the other hand, hyperactive antifreeze proteins bind to both primary prism and basal planes providing greater ice crystal coverage [17]. Consequently, extensive coverage inhibits ice growth from all angles leading to greater thermal hysteresis activity.

Uncontrollable ice burst between moderate and hyperactive antifreeze proteins differ in the direction of growth. Hyperactive bursting is speculated to be more damaging due to greater ice crystal coverage where explosive growth can occur following a small defect in coverage [30]. Hyperactive antifreeze proteins have weaker binding at the primary prism plane; thus, hyperactive ice bursts tend to occur along all six primary prism planes. Moderate protein leaves the basal plane uncovered hence leading to moderate ice bursts at the two basal planes. Less uncontrolled growth leads to lower damage. Consequently, major differences between moderate and hyperactive proteins occur due to differential plane binding.

#### 1.2.3. Ice Recrystallization Inhibition

**Figure 5:**
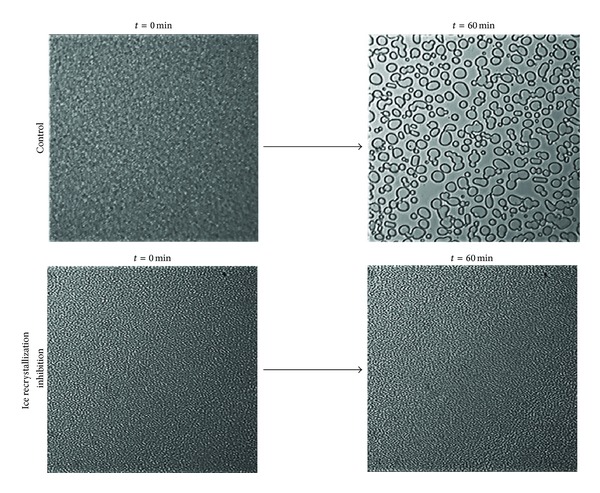
Ice recrystallization inhibition by antifreeze proteins. Flash frozen samples are held at −6°C for 60 minutes. Following this wait, ice crystal size at both time points are compared. Top figures show large recrystallized ice using a control solution, consisting of 50 mM Tris (pH 8.0) and 50 mM NaCl. Bottom figures show ice recrystallization inhibition using an antifreeze protein solution containing 0.1 mg/mL AfpA in control buffer.

At high subzero temperatures, smaller ice crystals recrystallize into larger ice crystals via the Kelvin effect [15]. This recrystallization structurally stabilizes ice by reducing the surface area exposed for melting [30]. The second antifreeze activity, ice recrystallization inhibition, prevents ice recombination by making smaller ice crystals more energetically favourable than larger ones (Figure 5). Using adsorption inhibition, Bound antifreeze proteins inhibit rapid water movement between ice crystals preventing destabilization of small ice crystal grains, thus minimizing ice recrystallization for ice crystal stability [30].

Unlike thermal hysteresis activity, ice recrystallization inhibition occurs at low concentrations of antifreeze proteins [1]. A concentration of 0.1 *μ*g/mL is sufficient for full ice recrystallization inhibition. This activity can even be enhanced with addition of ammonium bicarbonate [30]. Unfortunately, current assays are at best semi-quantitative, relying on visual observation and susceptible to inaccuracies in interpretation [18]. Consequently, this activity is typically reported as an estimate.

#### 1.2.4. Antifreeze Protein Classifications

There has been a continuing debate on the classification of a variety of antifreeze proteins. For a start, both thermal hysteresis and ice recrystallization inhibition activities are considered to be required for classification as antifreeze proteins. However, Middleton and colleagues [29] have suggested reclassifying proteins with mainly ice recrystallization inhibition and slight thermal hysteresis activity as ice binding proteins. This reclassification is problematic and would affect the majority of plant and bacterial antifreeze proteins. Furthermore, ice binding is not limited to antifreeze proteins; ice nucleation proteins also bind to ice to exert their effects [31].

At times, a protein may have only one of the two antifreeze activities. It was proposed that proteins with exclusively thermal hysteresis activity be reclassified as thermal hysteresis proteins [32]. Conversely, there have been reports of proteins that act exclusively in ice recrystallization inhibition [1]; it has been proposed that these proteins should be reclassified as ice structuring proteins. Due to their great variation, definitive classification of antifreeze proteins remains elusive.

#### 1.2.5. Freeze Avoidance and Freeze Tolerance

The variation in the strength and type of antifreeze activity is reflective of an organism's survival strategy. Generally, there are two strategies: freeze avoidance and freeze tolerance. A freeze avoidance strategy relies on preventing the ambient temperature reaching below freezing temperatures. Typically, this survival strategy is associated with either environments with low temperature fluctuations or mobile organisms such as fish and insects [21, 29]. These organisms depend on high thermal hysteresis activity where a greater freezing point depression is enough to encompass the temperature range of the environment. Consequently, frost damage is avoided.

Freeze tolerance is a strategy that involves minimizing inevitable frost damage to organisms [8]. It is commonly associated with environments with high subzero temperature fluctuations and immobile organisms such as plants and microbes [15, 29]. In these conditions, expressed antifreeze proteins are characterized by low thermal hysteresis activity while focusing on ice recrystallization inhibition. Low thermal hysteresis activity minimizes uncontrollable ice growth and frost damage when ambient temperature reaches the freezing endpoint [22, 28]. However, when temperature fluctuates to high subzero temperatures, ice recrystallization inhibition protect against freeze-thaw stress [15]. With these antifreeze proteins, inevitable frost is maintained as small, non-lethal ice crystals. Overall, different organisms and their surroundings influence the strength and type of antifreeze proteins expressed as well as survival strategy used.

#### 1.2.6. Ice Structuring and Shaping

**Figure 6:**
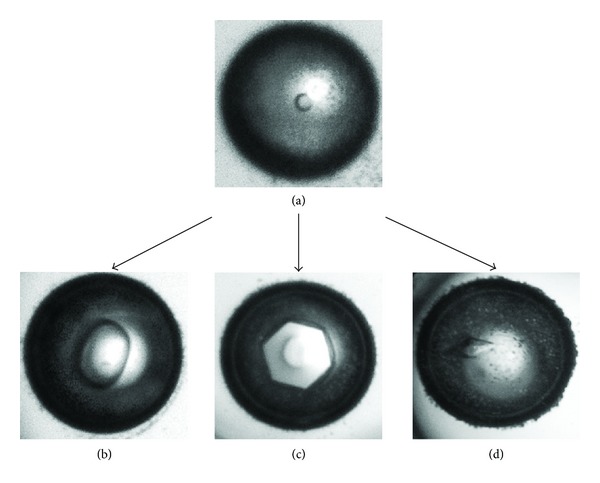
Common ice crystal morphology changes based antifreeze activity present. (a) Prior to ice crystal shaping, ice crystals are a spherical shape. (b) When there is a lack of ice crystal shaping, the crystal grows and remains circular (control buffer only). (c) Low level of antifreeze activity is represented by a hexagonal ice crystals. (d) Ice crystals seen with moderate level of antifreeze activity are usually observed as hexagonal bipyramids.

Ice morphology changes depending on surrounding molecules in the environment. As a byproduct of adsorption inhibition, antifreeze proteins have been documented to alter ice morphology in its supercooled state [1, 4, 24, 33]. Morphology shaping is dependent on the protein bound ice crystal plane. Typically, adsorption to the primary prism plane shapes the ice crystal into either a hexagonal or hexagonal bipyramid, as shown in Figure 6 using an antifreeze protein from *Pseudomonas putida* GR12-2. Antifreeze proteins found to bind both the primary prism and basal planes can produce other shapes such as a hexagonal crystal plate or a lemon shape [15, 22, 26]. Unusually, bacterial antifreeze proteins *Mp*AFP and *fl*AFP, from *Marinomonas primoryensis* and *Flavobacterium xanthum*, were found to lack ice structuring capabilities [21, 34].

#### 1.2.7. Bacterial Antifreeze Proteins

Within the last two decades, antifreeze proteins have been found in variety of bacteria from cold habitats [4, 6, 14, 18, 34, 35]. However, for the majority of these instances, only basic characterization of the proteins has been documented. Hypothesized to use a freeze tolerance strategy, these bacteria are commonly reported with low thermal hysteresis values with ice recrystallization inhibition activity. Consequently, activity detection studies on bacterial antifreeze activity focus on ice recrystallization inhibition and ice morphology shaping [15, 36].

The first documented bacterial antifreeze activities were found in the psychrophile *Micrococcus cryophilus *and the soil bacterium *Rhodococcus erythropolis *[37]. Thermal hysteresis values were reported as 0.29°C and 0.35°C, respectively. Following protease degradation and subsequent activity loss, this activity was attributed to a protein source. Unfortunately, detailed characterization of these AFPs was not reported. However, the first isolated bacterial antifreeze protein was documented in *Pseudomonas putida *GR12-2 [4]. This bacterium, originally isolated from the Canadian Arctic, is capable of proliferating at low temperatures (i.e., 5°C) and able to survive freezing temperatures, −20°C and −50°C, without the aid of cryoprotectants. When antifreeze activity was assessed, a moderately low thermal hysteresis value of 0.11°C was observed; the ice crystals were also shaped into a hexagonal bipyramids. This activity was determined to be from a protein when proteinase, heat, and chemical treatments eliminated activity.

The antifreeze protein, AfpA, isolated from *Pseudomonas putida *GR12-2, was reported to be 164 kDa in size with both sugar and lipid moieties [7]. Muryoi and colleagues [11] were able to isolate the gene encoding this AFP and determine its primary protein sequence. From these data, 7 N-glycosylation, 2 O-glycosylation, and 20 myristolation sites were predicted. Although other antifreeze proteins have been observed to be posttranslationally modified, typically with glycans, such heavy modifications are unusual, particularly for a bacterium [3, 9]. Following chemical deglycosylation, Xu and colleagues [7] discovered that at least 72 kDa of the original 164 kDa antifreeze protein, AfpA, is composed of glycans. Furthermore, the amount of lipidation has not been determined. Therefore, the percentage of protein in this antifreeze protein is not known and requires further work on both structure and role of these complex modifications.

A *Moraxella* sp. strain from Antarctica was also found to have antifreeze activity [35]. A 52 kDa lipoprotein was found to alter ice crystals into a hexagonal shape. This protein had a maximal thermal hysteresis value of approximately 0.2°C at a concentration of 1.5 mg/mL. Since then, in samples taken from Antarctic lakes, Gilbert and colleagues [18] have identified 186 bacterial isolates with ice recrystallization inhibition activity using a high throughput AFP protocol. Opaque solutions, containing small and dense ice crystals, indicated ice recrystallization inhibition activity. Unfortunately, this assay is largely qualitative and is very subjective; therefore, relative levels of this activity are not known.

Out of these 186 isolates, 19 of them were found to have thermal hysteresis activity, notably *Marinomonas protea*, reclassified as *Marinomonas primoryensis *[21]. This bacterium expresses a large hyperactive protein (>1000 kDa), *Mp*AFP, with a thermal hysteresis activity of approximately 2°C at 0.1 mg/mL [28]. At similar concentrations, a moderate fish antifreeze protein has an activity of 0.1°C. Furthermore, an explosive burst corresponding to uncontrollable ice growth parallel to the *α*-axes along with visualized binding to both primary prism and basal planes confirms the hyperactivity of this antifreeze protein [17, 21]. Unusually, this bacterial antifreeze protein lacks ice crystal structuring abilities despite its adsorption to both common ice planes.

Unlike other bacteria, a high thermal hysteresis activity suggests a freeze avoidance survival strategy for *Marinomonas primoryensis*. Moreover, this bacterium lives in a thermally buffered lake where temperatures are maintained between −1°C and 1°C in temperature consistent with a freeze avoidance strategy [18]. However, antifreeze activity is lost when the metal chelator, EDTA, removes calcium ions from the antifreeze protein [21]. This calcium dependency was confirmed after solving the structure of the antifreeze protein [17]. The availability of these ions becomes important and is influenced by localization of the active protein within the bacterium. Initially, the *Mp*AFP was thought to be localized in either the cytoplasm or periplasm of the bacterium [21]; however, a recent study by Guo and colleagues [38] reports that the antifreeze protein is located on the cell surface. These researchers have suggested that this localization is required for the transient binding the bacterium to ice to ensure better access to nutritional resources. Currently, *Mp*AFP is considered to be an anomaly amongst bacterial antifreeze proteins and closely resembles insect antifreeze proteins instead.

One of the more recently discovered bacterial antifreeze protein is from the bacterium *Flavobacterium xanthum *[34]. A cell free extract at 0.7 mg/mL was found with a thermal hysteresis activity of 0.04°C. In this case using size exclusion purification, a 59 kDa antifreeze protein was isolated. This cytoplasmic protein was found to have higher ice recrystallization inhibition activity than AfpA and, similar to *Mp*AFP, it lacked distinct ice shaping capabilities. Unusually, the protein can be activated by the addition of 0.5 M malate, reaching up to 5.2°C in thermal hysteresis activity.

Notwithstanding the fact that it has been 20 years since the first bacterial antifreeze was reported, there are relatively few detailed studies dealing with bacterial antifreezes. Nevertheless, there are an increasing number of reports suggesting the more widespread presence of bacterial antifreeze proteins. For example, *Pseudomonas *sp. UW4, a plant growth-promoting bacterium originally isolated from the rhizosphere of reeds growing on the campus of the University of Waterloo in Ontario, Canada [39], was speculated to express antifreeze proteins when extracts from this bacterium facilitated the formation of ice crystals into non-circular shapes [33]. Other bacteria observed to have ice recrystallization inhibition activity include* Sphingomonas* sp., *Halomonas* sp., *Pseudoalteromonas* sp., *Stenotrophomonas maltophilia*, *Psychobacter *sp., *Enterobacter agglomerans*, *Pseudomonas fluorescens, Rahnella *sp., *Duganella zoogloeoides*, *Erwinia billingiae*, and *Sphingobacterium kitahiroshimense* [1, 40]. Wilson and Walker [6] and Wilson et al. [41] have reported additional bacterial genera with ice recrystallization inhibition activity, but the source of the activity remains to be confirmed as antifreeze proteins; these include *Acinetobacter*, *Bacillus*, *Buttiauxella*, *Chryseobacterium, *and *Idiomarina*. Clearly, with so many different bacteria exhibiting antifreeze activity, there are many unanswered questions.

### 1.3. Bacterial Ice Nucleation Proteins

Ice nucleation proteins are characterized by their ability to initiate heterogeneous ice crystallization at high subzero temperatures up to −2°C [1]. These large proteins utilize their ice binding site(s) to provide an ice lattice template for the ordering of free water molecules [7] with the consequence that the ordered water molecules become ice crystal nuclei.

Ice nucleation activities have been documented in numerous ice nucleation active bacteria [1]. These bacteria are commonly gram negative, epiphytic, and pathogenic. Furthermore, they may either be psychrophilic or mesophilic.  Table 1 outlines the bacteria with confirmed or associated ice nucleation activity. Most of these bacterial ice nucleation proteins have not been well characterized at the protein level. Unlike most bacteria, *Pseudomonas syringae *has been documented to produce several variants of ice nucleation proteins (i.e., InaK, InaQ, InaV, and InaZ) and has been studied extensively [31, 42].

Xu and colleagues [7] speculate that ice nucleation proteins assist the survival of the bacteria that synthesize them. Following ATP-dependent transport to the outer membrane, it is argued that ice nucleation proteins direct ice formation into the extracellular space [7, 42]. This control of ice crystal localization provides time for the organism to physiologically adapt to freezing stress.

Due to an inability to measure directly ice nuclei formed, ice nucleation activity is reported as the temperature required to freeze 50% of the droplets (T_50_) added to a thermoelectric cold plate [1]. Recently, Hartmann and coworkers [43] have argued that this method inaccurately measures ice nucleation activity since each droplet varies in the number of ice nuclei present and does not account for ice nucleation by substrates in solutions. As an alternative, this research group has developed a model that measures ice nucleation rate assuming that ice nucleation complexes in droplets are Poisson distributed. However, whether this model becomes the new standard for this measurement remains to be determined. For the purposes of this review, ice nucleation activity is reported as T_50_ temperatures. Reported values have revealed that ice nucleation proteins vary greatly in activity level depending on protein composition. Consequently, these proteins can be grouped into three classes [44].

Class C (type III) ice nucleation proteins have the weakest activity, where T_50_ is less than −8.0°C [1]. For example, *Pseudomonas fluorescens* KUAF-68 and *Flavobacterium* sp. GL7 were classified as Class C activity with T_50_ values of −10.6 and −8°C, respectively [14, 15]. This class is composed of protein aggregates that can have an overall molecular weight greater than 1000 kDa [44]. Composed of glycoprotein aggregates, Class B (type II) ice nucleation proteins have moderate activity with a T_50_ value around −4.5°C. More specifically, these glycoproteins have been found to involve glucosamine and mannose modifications [44]. The most active ice nucleation proteins belong to Class A (type I), composed of lipoglycoprotein aggregates that are anchored to cell membranes via phosphatidylinositol, with T_50_ values up to −2°C [44, 45]. One of the strongest ice nucleation proteins, from *Pseudomonas syringae*, was reported with ice nucleation activity of −2°C [46]. Another class A nucleator was found in *Pseudomonas borealis *DL7 with an activity of −3.7°C [15]. Upon further investigation, the psychrophile *Pseudomonas borealis* produced an ice nucleation protein, *Pb*INP [13, 47]. When the gene encoding this protein was expressed in *E. coli*, the recombinant *Pb*INP had a mean freezing temperature of −5°C. This result indicates that there is some variation between the native ice nucleator and its measured activity and its recombinant equivalent.

According to this class model, posttranslational modifications such as glycosylation and lipidation can enhance ice nucleation activity. Muryoi and colleagues [11] were able to confirm that glycosylation plays a role in ice nucleation. After deglycosylation of AfpA from *Pseudomonas putida* GR12-2, a decrease in ice nucleation activity was observed. However, the direct role and magnitude of these modifications remain unclear.

#### 1.3.1. Extracellular Ice Nucleating Material

Some ice active bacteria exert ice nucleation activity via secreted ice-like liposomes known as extracellular ice nucleating material [48, 49]. These bacteria include *Erwinia carotovora*, *Erwinia herbicola*, *Erwinia uredovora, *and two strains of *Pseudomonas fluorescens *[48–52]. When Kawahara and coworkers [49] examined the ice nucleating spectra of the cell free ice nuclei from *Erwinia uredovora*, they found three distinct ice nucleation patterns matching the three classes of ice nucleation proteins. Also, these ice nucleation complexes were composed of 43% protein, 35% polysaccharide, 12% polyamine, and 10% lipid. Based on these observations, these researchers concluded that some ice active bacteria secrete liposomes with embedded ice nucleation proteins rather than anchoring the proteins to its cell membrane.

### 1.4. Simultaneous Antifreeze and Ice Nucleation Activity

Some bacteria have been reported to have both antifreeze and ice nucleation activity; this includes strains such as *Pseudomonas fluorescens* KUAF-68 and *Pseudomonas borealis *DL7 [14, 15]. Cooperatively, these two activities have been speculated to enhance the freeze tolerance survival of bacteria. Xu et al. [7] hypothesized that after directing ice nucleation externally, antifreeze proteins maintain small ice crystals with ice recrystallization inhibition protecting against freeze-thaw stress. Also, the low thermal hysteresis value minimizes damage from explosive ice crystal growth. More recently, Kawahara and colleagues [14] speculated that antifreeze proteins with minimal thermal hysteresis activity stabilize the outer membrane, while ice nucleation proteins minimize the supercooling point. However, the influence of both activities on freezing survival needs to be further investigated.

Interestingly, some ice crystal controlling proteins show both antifreeze and ice nucleation activities, while other ice crystal controlling proteins are exclusively one or the other of the two activities. For instance, *Pseudomonas putida* GR12-2 expresses AfpA, an antifreeze protein with a weak thermal hysteresis activity of 0.11°C and a class C ice nucleation activity of −11°C [4, 11]. In another case, an ice nucleation protein from *Xanthomonas campestris *exhibited ice recrystallization inhibition [53]. Furthermore, this protein was able to influence ice crystal growth following ice nucleation. At high concentrations of protein, Nada et al. [53] found a decreased ice growth rate along the *c*-axis and an increased ice growth rate along the *α*-axes. On the other hand, *Pseudomonas fluorescens *KUAF-68 was identified to express an 80 kDa antifreeze protein and a >120 kDa ice nucleation protein [14].

Recently, both ice crystal controlling activities have been theorized to utilize the same ice binding mechanisms. Initially, differential folding was suggested to be responsible for difference in activities. The latest hypothesis suggests that molecular size of the protein complex (affected by aggregation) influences the protein's functioning [7, 10, 13]. Large protein complexes, such as protein aggregates (e.g., the 600 kDa protein from *Pseudomonas fluorescens* KUAF-68, composed of 120 kDa monomers), usually exhibit ice nucleation activity [14]. Smaller molecular weight proteins, generally lower than 50 kDa, more typically exhibit antifreeze activity [13].

Strengthening this theory, Du and colleagues [12] observed that when type III antifreeze protein exceeds a critical aggregation concentration (CAC), the protein complexes showed ice nucleation activity. They hypothesized that at the CAC antifreeze proteins are optimally packed and adsorbed to ice crystals. Beyond the CAC, excess monomers are entropically driven to oligomerize leaving exposed ice binding sites to become ice nucleators, hence ice nucleation activity. However, antifreeze activity still may be present as new ice nuclei provide new interaction sites for monomeric antifreeze proteins beyond the CAC [12]. This provides an explanation for some bacterial antifreeze proteins that are large yet reported to have antifreeze activity such as AfpA [11]. Overall, protein molecular weight plays a major role in differentiating the two ice crystal controlling activities.

## 2. Ecology

With up to 80 to 85% of the planet's biosphere currently experiencing temperatures of 15°C or lower, many organisms have developed strategies against cold stress [5, 6]. Some adaptations protect against cell membrane rigidity, dehydration, desiccation, nutrient limitation, and more [6, 54]. The ability to express ice crystal controlling proteins is likely distributed globally [5, 15].

### 2.1. Aquatic Environments

Representing 71% of Earth's biosphere, the deep sea is the home to many psychrophilic and piezophilic microbes [5]. Psychrophiles are capable of growth and reproduction in cold temperatures, ranging from −15°C to +10°C, while piezophiles are organisms that thrive at high pressures. Surprisingly, these microbes have yet to be thoroughly investigated for ice crystal controlling activity. However, there was one case where bacterial isolates entrapped in Antarctic or arctic sea-ice either lacked or have only weak ice nucleation activity. Junge and Swanson [55] speculate such weak activity prevents ice formation on the cell by avoiding attachment to sea ice. Consequently, the observed cold tolerance of these microbes is speculated to be due to difference in cell membrane composition, cryoprotectant production, and sporulation.

Despite representing a small proportion of the biosphere, a number of microbial isolates from in and around lakes that express antifreeze activity have been identified [18, 41]. With arctic and alpine lakes, seasonal fluctuations in temperature present desiccation and freeze-thaw stress to inhabitants. These conditions would ideally select for ice active bacteria.

The lakes of Antarctica vary in salinity with time dependent fluctuation of ice formation. Ice coverage of these lakes influences water availability for enzymatic activity, which is a constant difficulty for microbial communities. Upon investigating 38 lakes in the Vestfold Hills of Eastern Antarctica, Gilbert and colleagues [18] discovered that some lakes were consistently thermally buffered between −1°C and 1°C at the ice water interface. Cold induction of antifreeze production at similar temperatures in both fishes and bacteria indicates that these lakes are an ideal location for ice active bacteria [7]. Antifreeze protein producing bacterial isolates were found to be primarily *γ*-proteobacteria with eleven isolates confirmed for ice recrystallization inhibition [18]. One isolate was identified as the reclassified antifreeze producing bacterium *Marinomonas primoryensis* [28]. In a separate study, the Antarctic bacterial isolate *Pseudomonas* KUIN-1 was also found to be ice active, expressing an ice nucleation and an unidentified cryoprotective protein as a protective response to high freezing stress [14].

Besides lakes, additional psychrophilic microorganisms have been found in other aquatic environments, such as glacial cores, and cryoconite holes [36, 56]. Common to these environments, pseudomonads have been shown to produce antifreeze and ice nucleation protein for ice binding [4, 15]. Other bacterial isolates from cryoconite holes were capable of producing antifreeze and ice nucleation activity as well. Therefore, other psychrophiles in these locations potentially have ice crystal controlling activity; however, further investigation is needed [5].

### 2.2. Terrestrial Environments

Soil bacteria communities differ from lake bacteria communities by adapting to harsher conditions such as high fluctuations of lower subzero temperatures [5, 21, 29, 36]. Within the soil from Ross Island in the McMurdo Dry Valley of Antarctica, Kawahara's research group [14] found 11 of 135 bacterial culturable isolates tested positive for ice nucleation activity. The most active strain was identified as a novel bacterial species and classified as *Pseudomonas antarctica*. In the same study, 6 out of 130 strains were capable of producing antifreeze activity. From these isolates, the greatest activity recorded was a thermal hysteresis activity of 0.08°C form a strain identified as *Moraxella*. A less active isolate, identified as *Pseudomonas fluorescens* KUAF-68, was reported with an activity of 0.03°C. The lower soil antifreeze activity (~0.1°C), compared to the significantly higher lake antifreeze activity (~6°C) from bacterium *Marinomonas primoryensis*, supports a freeze tolerance strategy for this Antarctic soil bacterial community.

Unlike Antarctic soils, arctic soils are warmer with greater water and nutrient bioavailability [5]. These arctic soils are primarily dominated by gram negative bacteria belonging to the *α*-, *β*-, and *γ*-proteobacteria class with pseudomonads representing 60% of all isolates in arctic soil. One such bacterium, *Pseudomonas putida *GR12-2, was found in soil of the Canadian High Arctic, where temperatures range from 5°C to 10°C during spring [4]. These temperatures are known to induce antifreeze protein expression permitting proliferation at low temperature [7]. Consequently, ice active bacteria inhabit arctic soils as well.

Permafrost is defined by consistent subzero temperatures persisting for two or more years [6]. Microbial soil communities in permafrost have been found to be resistant to freeze-thaw stress, radiation, and a wide range of antibiotics [5]. Resistance to freeze-thaw stress suggests these microbes harbor ice crystal controlling activity. *Exiguobacterium* and *Psychrobacter* isolates, from Siberian ancient permafrost, were shown to have ice nucleation activity and cell membrane composition suitable for fluidity under subzero temperatures. Therefore, other soil psychrophiles from permafrost potentially express ice crystal controlling proteins.

At high mountain altitudes, microbial communities in alpine soil endure high temperature fluctuations, high precipitation, and freeze-thaw stress [5]. Consequently, psychrophiles found in alpine soil have a large potential to express ice crystal controlling proteins. At lower altitudes, microbial communities experience very quick freeze-thaw events [36]. Chinook winds in Canada have been reported to increase temperatures by 20°C in an hour. This fluctuation can create a freeze-thaw cycle ranging from −35°C to 35°C. To investigate freeze-thaw resistance, Walker and colleagues [36] mimicked these conditions by exposing soil samples to 48 freeze-thaw cycles between −18 and 5°C. Overall, decreases in abundance, diversity, and diameter of surviving bacterial isolates were seen, potentially due to diverting resources to maintain physiology instead of growth.

After thermocycling, the most viable isolates were gram negative *Buttiauxella *or *Chryseobacterium, *while the less viable gram positives were either *Acinetobacter *or *Enterococcus *isolates [36]. Interestingly, in a follow-up study, Wilson et al. [15] isolated bacteria in these soil samples through ice affinity binding and found the dominant isolate to belong to the *Paenibacillus* genus, a gram positive bacterium. This suggests that production of ice binding proteins may not be the dominant strategy for freeze-thaw survival. However, Wilson and colleagues [15] reasoned that microbial interactions within the community compensates for bacteria with lower cryoprotection. When *Chryseobacterium* sp. strain C14, an isolate found with ice recrystallization inhibition activity, was inoculated with *Enterococcus *sp. strain C8, viability of the latter strain decreased by three rather than the original six orders of magnitude after several freeze-thaw cycles. The researchers suggested that commensalism between the two bacteria by secretion of antifreeze protein enhances the survival of the overall microbial community. However, sharing ice crystal controlling proteins may not be sufficient for freeze-thaw survival of a microbial community. Biofilm production from *Erwinia billingiae* and *Sphingobacterium kitahiroshimense* was found to assist freeze-thaw survival of the ice nucleation active bacterium *Pseudomonas syringae *[40]. Thus, the sharing of ice crystal controlling protein is one of many factors contributing to the freeze-thaw survival of microbial community as a whole.

In temperate soils surrounding lakes, bacteria have been reported to have antifreeze activity [41]. Despite warm average temperatures, seasonal fluctuations can promote the expression of ice binding proteins [25]. Wilson and colleagues [41] investigated soil bacteria at two lakes. Daring lake in the Northwest Territories, Canada, endures long, harsh winters and great temperature fluctuations during spring. Temperate Gould Lake in Watershed, Ontario, Canada, endures lower seasonal temperature fluctuations. Similar to a previous study by this research group, simulated freeze-thaw cycles revealed reduced abundance and diversity of the original microbial community. Comparatively, Gould Lake samples displayed a greater loss of diversity but ended equally as diverse as Daring Lake samples. The surviving isolates were originally low in abundance for both samples; however, these freeze-thaw cycles selected for bacterial isolates with cryoprotective abilities [41]. Dominant genera were *Bacillus *and *Pseudomonas *for Daring and Gould Lake samples respectively.

Not all isolates revealed ice crystal controlling activity; however, Gould Lake isolates *Bacillus *sp. strain G1a1, *Buttiauxella *sp. strain G2b1, and *Enterobacteriaceae* sp. strain G3b1 along with Daring Lake isolates *Chryseobacterium piscium *DL11 and *Pseudomonas *sp. strain DL13 displayed ice recrystallization inhibition activity equivalent to type III antifreeze proteins [41]. Furthermore, *Bacillus* sp. strain G1a1 had type I ice nucleation activity, while *Buttiauxella* sp. strain G2b1 showed ice structuring ability. However, when bacteria were isolated on the basis of their ice affinity, different bacterial genera were recovered including strains of *Pseudomonas*, *Stenotrophomonas*, *Chryseobacterium*, *Flavobacterium, *and *Acinetobacter* [15]. These differences can be influenced by the cellular localization of the expressed ice binding proteins; thus, ice affinity binding may not isolate cytoplasmic or periplasmic expressed proteins [21]. From these bacterial isolates, *Pseudomonas borealis *DL7 and *Flavobacterium *sp. strain GL7 were determined to have type I and II ice nucleation activity, respectively. Furthermore, *Chryseobacterium *sp. strain GL8 and *Acinetobacter radioresistens* DL5 were tentatively classified as having similar ice recrystallization activity as *Chryseobacterium* sp. strain C14 [15].

The inconsistency in antifreeze activity, ice nucleation activity, and freeze-thaw survival indicates that these bacteria possess a complex array of mechanisms for cold adaptation with no single dominant mechanism. Tentatively, it appears that a combination of many different adaptations and commensalisms between isolates are sufficient for cold stress survival.

### 2.3. Other Environments

#### 2.3.1. Plants

Ice nucleation active bacteria have been commonly found on plant surfaces [46]. However, the ice active phenotype of these bacteria is sensitive to environmental changes such as light intensity and ambient humidity [57, 58]. Cambours et al. [57] reported that ice active epiphytes are prevalent in the spring whereas ice active endophytes are more common during fall. Also, the ice active phenotype is dependent not on pathogenicity, but on the strain and host plant genotype [58]. One common epiphytic bacterium, *Pseudomonas syringae*, is usually pathogenic to plants causing foliar necrosis in host plants [31]. The bacterium uses ice crystal formation to injure and stress plants in order to facilitate subsequent bacterial infection [59]. When ice nucleation active bacteria populations are decreased, frost injury to plants is reduced. Consequently, frost injury to frost sensitive plants and the presence of ice active bacteria are closely correlated. The rhizospheres of cold-inhabiting plants are also home to ice active microbes, such as *Pseudomonas *sp. UW4 isolated from common reeds in Waterloo, Ontario [33]. Other soil bacteria are exposed to seasonal fluctuation and frost damage and thus potentially ice active microbes [15].

#### 2.3.2. Insect Gut

Within insects, ice nucleation active bacteria have been isolated from indigenous gut microflora [60]. These bacteria are believed to affect the freezing temperature of insect bodily fluids because when ice nucleation active bacteria were introduced via ingestion, the cold tolerance of insects was reduced [61, 62]. However, upon isolating the ice nucleating bacterium *Kluyvera* sp., Nicolai and coworkers [60] found a weak ice nucleation activity of −9°C. Although this bacterium is ice nucleation active, this research group concludes that the ice nucleation activity of gut microbes is not involved in increasing insect susceptibility to freezing and may play a different role.

#### 2.3.3. Atmosphere

Viable microorganisms found in the atmosphere, up to the stratosphere, must endure temperatures reaching −100°C, UV radiation, oxidative stress, low nutrient availability, and desiccation [5]. These conditions can trigger and influence ice nucleation activity in some ice active microbes [63]. UV-C and not UV-A radiation have been shown to reduce ice nucleation activity [64, 65]. In addition, acidic pH in the atmosphere can also decrease ice nucleation activity. The remaining ice nucleation activity from these microbes can seed cloud condensation and formation, as observed in controlled atmospheric chambers [66]. Acting as ice forming nuclei, these bacteria also catalyze ice crystal formation in clouds leading to precipitation. Consequently, ice active bacteria have been found to be more prevalent in rain than in surrounding air [67]. Furthermore, indigenous ice nucleation active populations of *Pseudomonas syringae* have been isolated from snow from a ski resort in Greece [68]. This suggests that these microbes play an active role in precipitation and snow formation. Joly and colleagues [69] confirmed ice nucleation activity after isolating 44 strains from cloud water. Out of these strains, seven were ice active bacteria; four were *Pseudomonas syringae*, two were *Xanthomonas *sp., and one was *Pseudoxanthomonas* sp. [69]. These strains were active at three different temperature ranges. *Pseudomonas syringae* were active at −4°C or above, while *Xanthomonas *sp. *and Pseudoxanthomonas* sp. were active at either between −4°C and −6°C or below −7°C. The prevalence of ice active bacteria in atmospheric clouds and the distribution of these strains by precipitation imply that ice active bacteria can be globally distributed through the water cycle.

### 2.4. Evolution

The traditional outlook of environmental selection for physiological traits has been speculated to apply to the emergence of ice crystal controlling protein for cold stress survival [6]. After applying 48 freeze-thaw cycles, all surviving bacterial isolates displayed cryoprotective activity, among them ice crystal controlling activity, suggesting freezing conditions select for cryoprotection. Similarly in Antarctic lakes, ice active bacterial selection is correlated with salinity where freshwater lakes showed no signs of ice active bacteria [18]. This research group speculated that the brackish and saline system is a major driving force in development of antifreeze proteins due to the high fluctuation in temperature of more saline lakes. This presents greater freezing stress and is a predominant selection factor.

Lin et al. [26] note that the structural diversity of antifreeze proteins across a wide variety of organisms suggests convergent evolution through environmental selection. Presumably, expression of antifreeze protein arose as numerous independent events.

The environment both affects and is affected by the evolution of antifreeze proteins. A study by Near and colleagues [70] demonstrates that the expressed ice crystal controlling protein selects for the specific environments by allowing organisms to expand into new niches.

#### 2.4.1. Gene Evolution

In many species, ice crystal controlling gene families have been reported to produce multiple protein isoforms, all of which contribute to overall activity. The ice nucleating bacterium, *Pseudomonas syringae* has a minimum of three ice nucleation protein isoforms, each with high similarity to one another between sequences [31, 42, 63]. Antifreeze gene families have been commonly found in fish and insects but not investigated in bacteria [23, 26, 71]. These gene families are believed to arise from gene duplication with each duplicated gene subsequently developing mutations. Multiple antifreeze protein isoforms are selected for greater ice crystal coverage leading to greater activity.

A characteristic of each antifreeze protein isoform is the repetition of certain amino acid sequences; this is speculated to arise from internal duplication of genes [23]. One antifreeze protein showed clear internal homology with 63% sequence identity in a 30 amino acid insertion, suggesting that the Thr-X-Thr ice binding motif is involved with antifreeze activity. Due to a correlation between the number of repeats and thermal hysteresis activity, selection of better ice crystal controlling proteins can arise from internal gene duplication [3].

## 3. Biochemistry

### 3.1. Ice Crystal Interaction

Ice crystal controlling activities require a unique protein-ice association. To bind ordered water molecules on the ice crystal surface, ice crystal controlling proteins utilize a flat, hydrophobic surface complementary to the atomic spacing of the ice crystal planes, mimicking an ice crystal surface. Variations in atomic spacing at the protein binding site influences the affinity and location of ice crystal binding (Figure 1) [29, 72]. Ice crystal controlling activity is affected to a significant extent by solvent exposed amino acid residues that are found along the ice-binding surface. By changing theses residues to match one of the ice crystal planes, inactive antifreeze protein isoforms can be altered to be active [73]. Consequently, solvent exposed residues play a major role in ice crystal interaction.

Antifreeze proteins have been proposed to inhibit ice growth through an adsorption inhibition mechanism based on the Kelvin effect [16]. According to the Gibb-Thompson equation, the addition of water molecules along the ice surface becomes energetically unfavourable after adsorption of antifreeze proteins. One model, the mattress model, hypothesizes that water molecules are continuously added in the limited space (~20 nm) between adsorbed antifreeze proteins [3, 74]. However, due to ice growth being perpendicular to the ice plane, water molecules form curved surfaces in this space. Upon reaching a maximum curvature, additional water molecules would increase the surface area to an unstable degree making it energetically unfavorable hence inhibiting further ice growth [75].

However, the adsorption inhibition mechanism has one major assumption: irreversible binding of antifreeze proteins [76]. The problem with reversible binding is that transient desorption of antifreeze can reveal new sites for energetically favourable additions of water molecules; this can lead to uncontrollable ice growth [74]. Furthermore, with a molar excess of water, a high concentration of antifreeze proteins is required to establish equilibrium between water and protein bound to ice. A colligative depression of 0.5°C would require a local concentration of 300 mg/mL. Supported by empirical evidence of non-colligative depression, antifreeze proteins are assumed to bind irreversibly.

However, Pertaya and colleagues [74] argue that antifreeze proteins use colligative temperature depression prior to reaching maximal thermal hysteresis activity. This suggests a temporary equilibrium exchange of bound and unbound antifreeze proteins. As a result, these researchers propose initial irreversibly bound antifreeze proteins are engulfed during ice growth and thus require additional antifreeze protein for further inhibition. Despite lacking evidence of detectable reversibility, assuming slow exchange is occurring, and using docking simulations the group concluded that reversible exchange is quite slow, taking more than a week. Therefore, antifreeze protein binding is essentially quasi-permanent. Thus, the current model of antifreeze binding proposes that adsorption to ice crystals occurs via similarity and complementary matching of atomic spaced water molecules in a quasi-permanent manner.

### 3.2. Antifreeze Protein Structure

The efficient inhibition of ice crystal growth requires a large variety of ice binding surfaces complementary to the many ice crystal planes. This diversity in binding can provide ideal coverage of the ice crystal [17]. Adapting these circumstances, convergent evolution has developed a structurally diverse class of proteins that can perform this function. These structures can range from single *α*-helices to large ordered *β*-solenoids [28, 77].

There are some similarities amongst antifreeze proteins. One similarity is a flat, hydrophobic ice binding surface that is a common antifreeze protein identifier. With this surface, antifreeze proteins may even be globular [78]. In order to maintain flatness in this surface, the antifreeze protein must maintain rigidity during ice binding [29]. Some antifreeze proteins have internal asparagine ladders, an external *α* -helix or extensive hydrogen bonding network in the protein core to retain rigidity [19, 29].

A second common feature amongst antifreeze proteins is the presence of amino acid tandem repeats in the protein sequence. These repeats are non-specific but are structured so that ice binding residues are aligned on one side to create an ice binding face [26, 28, 29, 76]. Furthermore, regularity of repeats creates order in the spacing between ice binding residues, which is essential to complement ice crystal planes. For example, to bind the primary prism planes, the distance between neighboring ice binding residues should be approximately 7.3 Ångstroms on one side and 4.5 Ångstroms on the perpendicular side. This distance creates a two-dimensional surface area with ordered ice binding residues spread in an array. Any greater difference of spacing in either direction can lead to improper complementary to ice, thus decreasing thermal hysteresis activity [29].

With these similarities in mind, Doxey et al. [79] were able to develop a antifreeze prediction program, AFPredictor. Another prediction program by Kandaswamy and colleagues [80], the machine learning AFP-pred, shows some promise of detecting important antifreeze peptide features. However, using a training set of sequences consisting of hypothetical and unconfirmed antifreeze proteins indicates that notwithstanding the value of these algorithms, further work is required for a more complete understanding of these proteins.

#### 3.2.1. Bacterial Antifreeze Protein Structure

Currently, the most well characterized bacterial antifreeze protein structure is *Mp*AFP from *Marinomonas primoryensis* [28]. *Mp*AFP consists of five distinct domains, of which two (Regions II and IV) contain repetitive sequences. Region II has 120 perfectly conserved 104 amino acid tandem repeats. Region IV is a smaller repeat region comprised of 13 tandem repeats of 19 amino acids each and was the domain confirmed with antifreeze activity.

**Figure 7:**
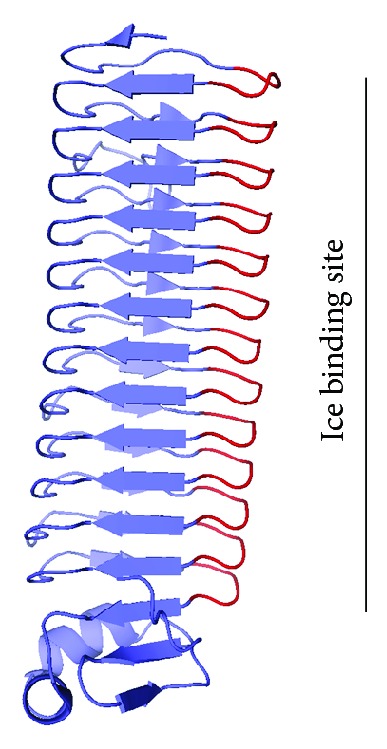
Protein structure of *Mp*AFP retrieved from RCSB Protein Data Bank (PDB). Ice binding site of the antifreeze protein lies along the aligned calcium binding turns, X-Gly-Thr-Gly-Asn-Asp. These calcium binding turns are highlighted in red using pyMOL.

Recently confirmed through X-ray crystallography, Region IV was modeled after alkaline phosphatase as a novel left handed *β*-roll [17, 28]. Each tandem repeat has a consensus sequence of X-Gly-Thr-Gly-Asn-Asp-X-U-X-U-Gly-Gly-X-U-X-Gly-X-U-X with X and U residues representing hydrophilic and hydrophobic residues, respectively [28]. Representing one *β*-roll loop each, these tandem repeats form a calcium binding turn along the length of the domain when X-Gly-Thr-Gly-Asn-Asp is aligned (Figure 7). In this case, the X residue is either an Ala or Gly residue. Calcium ions bound on all 13 loops stabilizes the main carbonyl of the first two Gly residues and a side carbonyl chain from the Asp residue in the X-Gly-Thr-Gly-Asn-Asp motif [17]. Each loop is also stabilized by the main carbonyls of Thr and hydrophilic X residues along with two side chain carbonyls by Asp residues in subsequent loops. Consequently, the calcium ion is locked into position differing only at the 13th loop. At the last loop, a Glu residue extends into the solvent to bind both a calcium ion and two water molecules. These calcium ions were confirmed to be an essential structural component of this protein [28]. When absent, the protein secondary structure is reduced to random coils.

These X-Gly-Thr-Gly-Asn-Asp turns were found to create a long and flat ice binding site (Figure 7). Solvent exposed Thr and Asx residues form an array down the length of the domain unlike the Thr-X-Thr ice binding motif found in insect antifreeze proteins [26, 28]. Exchanging the third Thr residue with Asx compensates pitch changes created by the bound calcium [17]. The two polar residues are separated by Gly residues thereby maintaining surface flatness while at the same time ordering oxygen atoms to form 7.4 Ångstrom by 4.6 Ångstrom long arrays. When solvent exposed Thr or Ser residues in this array were mutagenized to large Tyr residues, a loss of 50% or greater of the thermal hysteresis activity was reported [28]. When other non-ice binding hydrophilic residues were mutagenized, there was no significant loss in antifreeze activity. Garnham and colleagues [17] suggest that Tyr residues disrupted the flatness of the ice binding surface leading to decreased function.

From the crystal structure, Garnham and colleagues [17] discovered anchored clathrate water (i.e., water molecules contained in an ice-like lattice) along the length of this ice binding face. These water molecules were anchored to troughs created via Gly residue hydrogen bonding with main chain nitrogen atoms and side chain hydroxyl groups of solvent exposed Thr residues. Other water molecules, on the Asx side of the Thr-Gly-Asn motif, are bound by nitrogen atoms from the protein's main chain and from side chain oxygen atoms, from Gly and Asx residues, respectively. The specific ordering of these water molecules are maintained by the relative hydrophobicity of the ice binding surface and are involved in ice binding.

Non-ice binding residues within the repeats assist in maintaining protein structure and rigidity [17]. Hydrophilic residues become solvent exposed, while hydrophobic residues sustain the protein core. A major disruption of the protein structure occurs when a hydrophobic residue is replaced by a charged, Arg, residue; this reduced the activity by 75%. Overall, *Mp*AFP has a protein structure very similar to insect antifreeze proteins following the reported Thr-X-Thr motif [26].

The second most characterized bacterial antifreeze protein is AfpA from *Pseudomonas putida* GR12-2. Based on the protein sequence, Muryoi et al. [11] found that the secreted protein lacks a conserved canonical N-terminal signal peptide. However, they speculate secretion may occur using either a hemolysin-like secretion or type V autotransportation system. Other features reported are three hemolysin type calcium binding repeats and five Gly-X-Gly-X-Asp calcium binding motifs. Similar to *Mp*AFP, AfpA may potentially bind to calcium ions using Gly-X-Gly-X-Asp calcium binding turns., With only two characterized bacterial antifreeze proteins, an understanding of these proteins is still quite limited.

### 3.3. Bacterial Ice Nucleation

Unlike antifreeze proteins, ice nucleation proteins are large and multimeric with subunits ranging from 120 to 150 kDa in size [1]. Typically, these hydrophilic proteins are found anchored to cell membrane surfaces. The structural distinctions between antifreeze and ice nucleation proteins remain unclear, especially with recent speculation that these proteins may utilize similar ice binding mechanisms [13]. Furthermore, one secreted antifreeze protein found with ice nucleation activity, AfpA, has blocks of sequence that are similar to the ice nucleation protein InaV [11].

As a structurally homologous class of proteins, most ice nucleation proteins are composed of three domains: a N-terminal domain, a central repeating domain (CRD), and a C terminal domain [1]. These proteins generally vary in amino acid sequence and sequence repetition in the CRD. Without a limit to repeat number, this variation has been exploited to present recombinant proteins on the surface of cell membranes [31].

The relatively hydrophobic and globular N-terminal domain, comprising 15% of the protein, is hypothesized to bind to lipids, polysaccharides, and other ice nucleation proteins [42]. Interaction between ice nucleation proteins and lipids, especially phospholipids, has been documented to greatly influence ice nucleation activity [45]. This association permits the protein to anchor itself into the cell membrane, allowing aggregation and organized assembly for increased activity [81]. When the cell membrane of *Pseudomonas syringae* was destabilized by ozone treatment, the resultant ice nucleation activity was lowered from −2.8°C to −7.3°C [82]. A similar effect occurred when Govindarajan and Lindow [81] treated *P. syringae *with lipases, detergents, and organic solvents. However, ice nucleation activity could be recovered with phospholipid supplements. Yu and colleagues [83] also demonstrated the importance of fatty acid on ice nucleation activity; when recombinant *Pantoea ananatis *ice nucleation protein was expressed in *Escherichia coli *missing FabH, a protein responsible for fatty acid elongation, there was reduced ice nucleation activity. This activity loss was also recoverable by overexpressing FabH.

Investigating the N-terminal domain specifically in InaQ, Li and colleagues [31] were able to confirm this domain was involved in transportation and anchoring of the protein. They tested the accessibility of surface immobilized protein after either truncating or lengthening at the domain's two transmembrane sequences. Truncating the first transmembrane motif resulted in a major loss of surface anchoring with 93% of the GFP tagged domain found in the cytoplasm. On the other hand, increased repetition of this motif led to improved surface immobilization. Potentially, the domain anchors via a mannose-phosphatidylinositol, either N or O linked to an Asp, Ser, and Thr residue [1].

As the bulk of the protein, the central repeating domain is proposed to be the site of ice interaction [1]. There are three levels of repeat fragments [42]. Composed of 48 residues, the largest fragments have the highest fidelity. In InaZ from *Pseudomonas syringae*, the consensus is Ala-Gln-Glu-Gly-Ser-Asn-Leu-Thr-Ala-Gly-Tyr-Gly-Ser-Thr-Gly-Thr-Ala-Gly-Ala-Asp-Ser-Ser-Leu-Ile-Ala-Gly-Tyr-Gly-Ser-Thr-Gln-Thr-Ser-Gly-Ser-Glu-Ser-Ser-Leu-Thr-Ala-Gly-Tyr-Gly-Ser-Thr-Gln-Thr. This fragment can be broken into three mid-fidelity fragments of 16 residues each. In *Pb*INP from *Pseudomonas borealis*, the consensus of these fragments is Gly-Tyr-Gly-Ser-Thr-X-Thr-Ala-X-X-X-Ser-X-Leu-X-Ala [13]. When these 16 residue repeats were characterized in an ice nucleation protein from *Xanthomonas campestris*, Kumaki and colleagues [84] found that these residues formed a circular loop with *β*-turns. These 16 residue fragments can be further broken down into two low fidelity octapeptides, which are speculated to influence the bends in CRD folding.

Two decades ago, Kajava and Lindow [42] proposed a model for InaZ with the CRD folding in a *β*-conformation with alternating *β*-strands and random coils linked by interpeptide bonds. They speculated that stacking of *β*-structures in a hairpin manner produced a rigid structure with alternating patterns of rectangular and trapezoidal structures. Unlimited aggregation was believed to occur by interdigitization of similar sides of another ice nucleation protein (i.e., rectangular structures docked with other rectangular structures). More recently, Graether and Jia [10] argue that ice nucleation proteins fold into a *β*-helix using a Thr-X-Thr motif for ice binding similar to antifreeze proteins. An ice nucleation protein from *X. campestris *was speculated to form a *β* helix structure based on 16-residue loops allowing the alignment of Thr-X-Thr motifs [84]. However, both research groups agree that the abundance of Thr and Ser residues resides in the middle of the ice binding surface [10, 42].

**Figure 8:**
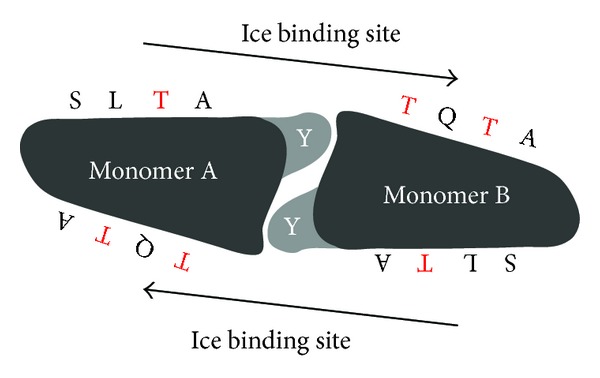
Schematic diagram of the dimerization of *Pb*INP using tyrosine ladders. Dimerization allows for tetrapeptides of each side of a monomer to create a relatively flat Thr-X-Thr based ice binding surface. Threonine residues used on the ice binding surface are highlighted in red. S: Ser, L: Leu, T: Thr, A: Ala, and Q: Gln. Adapted from Garnham et al. [13].

Further supporting Graether and Jia's [10] hypothesis, Garnham et al. [13] modeled *Pb*INP as a right handed *β*-helical structure that is stabilized by inward facing Ser and Gly ladders. The protein dimerizes via desolvation of the aromatic side chains from an exposed Tyr ladder on each monomer (Figure 8). Dimerization creates a flat ice binding surface by aligning side one of monomer A with side two of monomer B along the length of the CRD. More precisely, the aligned tetrapeptides per side create an octapeptide (i.e., Ser-Leu-Thr-Ala and Thr-Gln-Thr-Ala) with a Thr-X-Thr motif speculated to ice bind in a manner similar to insect antifreeze proteins [13, 17, 26]. Despite the overall hydrophilicity of the protein, these flat surfaces are relatively hydrophobic with each side of the dimer proposed as an ice binding surface for ice nucleation proteins.

In the last 15% of the protein, the function of the globular C-terminal domain remains undetermined and unexplored [42]. Li et al. [31] were able to confirm that the domain is not associated with either the cell membrane or cellular transport. When GFP tagged, the expressed domain remained in the cytoplasm. Conclusively, the N-terminal domain is responsible for transportation and anchoring, the CRD is responsible for ice interaction, and the function of the C-terminal domain function requires further study.

### 3.4. Ice Binding Mechanism

Ice binding depends on the ability to recognize and interact with ice crystal planes. Typically, ice crystal controlling proteins are comprised of a flat, hydrophobic ice binding surface [3, 29, 72, 77]. The arrangement of polar and non-polar residues on this surface needs to match the atomic spacing of water molecules on ice crystal surfaces for adsorption [2]. Furthermore, complementarity between protein and ice atomic spacing must be significant to prevent dimerization of antifreeze protein along the ice binding surface [85]. Non-binding regions of proteins are arranged for unfavourable water interaction to prevent engulfment by growing or neighbouring ice crystals [72, 74].

Currently, a detailed mechanism for adsorption inhibition is continuously evolving. Initially, hydrogen binding between the complementary ice binding surface and ice crystal planes was hypothesized to arrest ice growth [17]. However, this postulated mechanism was insufficient in explaining the high adsorption rate explicitly to ice crystals instead of excess water molecules in the system. Furthermore, one study found that the loss of a methyl group had a greater detrimental impact on thermal hysteresis activity than the loss of a hydroxyl group [76]. Regarding the loss of a hydroxyl group, a mutation from Thr to Ser led to loss of 90% of activity, while the latter condition, a mutation from Thr to Val, reported only a loss of 15% of activity. Therefore, hydrogen bonding may be necessary but not sufficiently for ice crystal binding.

A secondary hypothesis relies on entropy, van der Waals forces, and hydrophobic interactions for ice binding. It has been speculated that non-anchored clathrate waters within the troughs of ice binding surface are released immediately prior to adsorption [17, 72]. To prevent the loss of configuration, it becomes more favourable for these proteins to adhere to ice crystals. This binding prevents water interaction with the hydrophobic core. However, a molecular dynamic study has found that some ordered clathrate waters are not released upon binding. Although this hypothesis explains the kinetics of protein-ice interaction, it does not address the specificity of ice binding.

**Figure 9:**
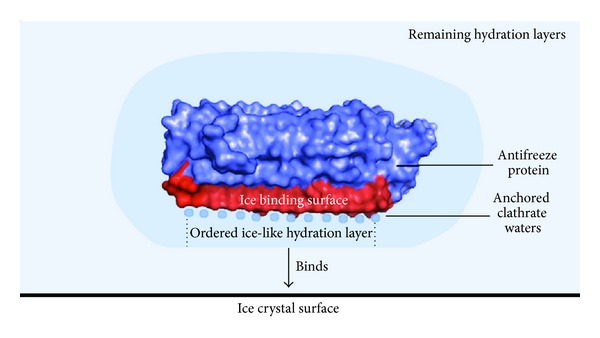
Schematic diagram of ice binding using an ice-like hydration layer (*Mp*AFP retrieved from RCSB Protein Data Bank). Anchored clathrate waters in the troughs of the ice binding surface orders the hydration layer to become ice-like. This hydration layer become an ice template allowing binding to ice crystal surfaces. The ice binding surface of the antifreeze protein is highlighted in red using pyMOL.

An alternate hypothesis proposes that anchored clathrate waters influence the hydration layers of the ice crystal controlling proteins [2]. These layers are speculated to influence the ice-water interface creating a binding site prior to ice crystal adsorption as shown in Figure 9 [2, 72]. When observed, hydration layers were tetrahedral and ice-like. However, disruption of the ice-like hydration layer by mutagenesis of increasingly bulky residues correlated with a loss of antifreeze activity [71]. Interestingly, although the ice-like qualities do not extend beyond the first hydration layer, it is believed to be sufficient in enhancing ice recognition. However, a recent study by Hakim and colleagues [85] suggests that extension of this ice-like property into additional layers allows antifreeze proteins to bind to more than one ice crystal plane.

Ice nucleation activity does not utilize the adsorption inhibition mechanism. However, Garnham and colleagues [13] speculate that ice nucleation also relies on anchored clathrate water. Tentatively, it may be speculated that ice nucleation proteins create an ice lattice within their hydration layers, similar to antifreeze protein. With the lack of ice crystals, water molecules can be ordered onto this lattice for embryonic ice crystal formation. Furthermore, structural homology of these proteins indicates that a single ice-like template is required only to initiate ice crystallization, whereas antifreeze proteins require a large diversity of ice binding surfaces to provide suitable coverage of existing ice crystals. Again, further investigation is required to fully understand the ice binding mechanism of ice crystal controlling proteins.

## 4. Gene Regulation

Knowledge of the regulation of ice crystal controlling protein expression is limited. Typically, these proteins are induced under cold stress and can be expressed seasonally [4, 7, 27]. Furthermore, Kawahara et al. [86] reported that antifreeze protein expression in *Pseudomonas putida *GR12-2 remained consistent following entry into the stationary phase, a growth stage associated with stress and cell maintenance. Similarly, ice nucleation proteins have also been reported to be expressed at high levels under conditions of cold temperature [87, 88]. In fact, Nemecek-Marshall et al. [87] shows that the highest expression in *Pseudomonas syringae *occurred from a combination of cold temperature and nutrient starvation, which is indicative of the stationary phase. However in another case, Chen and coworkers [88] demonstrated that *Pseudomonas fluorescens *MACK-4 showed optimal expression following cold temperature growth with sorbitol, mannose or starch, and peptone. This suggests that ice crystal controlling protein expression may be dependent on the bacterium studied, with cold temperature being a common inducer. Understandably, cold expression is required as some ice crystal controlling proteins have been reported to be thermolabile, becoming inoperable at regular bacterial growth temperatures [26].

However, this temperature-based induction is not very well understood. One model proposes that ice crystal controlling protein induction occurs as a response to upregulation of cryoprotective genes by a cold sensor [5]. A cold sensor located on the cell membrane is linked to a membrane-associated protein [89]. When the temperature decreases, the increasing rigidity of the cell membrane mechanically activates the cold sensor and subsequently the membrane-associated protein. A signal is passed on to the response regulator which ultimately upregulates cryoprotective genes without specificity. Amongst these genes are antifreeze and ice nucleation genes.

Regulation of ice crystal controlling proteins also occurs at the posttranslational level. N-terminal acetylation and/or C-terminal amidation have been found for some ice crystal controlling proteins [27]. In other instances, glycosylation and lipidation have been reported, especially in ice nucleation proteins [11]. Without such modifications, the cryoprotective activity of these ice crystal controlling protein is typically diminished. Consequently, the data suggest that regulation of the cryoprotective activity of ice crystal controlling proteins can occur at both the transcriptional and posttranslational level.

## 5. Conclusions

Antifreeze and ice nucleation protein production is a common strategy for the survival of various organisms in cold environments. The diversity between organisms suggests that due to convergent evolution the antifreeze protein expressed in a particular system may be unique to that organism or environment. Regardless, the ice crystal controlling nature of these proteins has been exploited in many biotechnical applications over the last two decades.

The most common application for antifreeze protein is the enhancement of cryopreservation of biological materials. Antifreeze protein treatments protect against freezing damage to cell membranes and their associated proteins by preventing large ice crystals or spicules development [90, 91]. In the medical field, these treatments have improved storage of oocytes and red blood cells as well as cryosurgery tissue preservation [90–92]. However, toxic levels of antifreeze proteins can induce intracellular ice formation leading to destruction of cells [93]. Within the food industry, antifreeze protein treatments aim to maintain food quality while increasing shelf life. Antifreeze protein injections reduced drip loss in frozen meats [94]. For frozen foods, antifreeze proteins help retain fermentation abilities of yeast in frozen dough and smooth texture in ice cream [95, 96].

Recently, antifreeze proteins have been considered for non-preservation based applications including use as hydrocarbon hydrate inhibitors and biopolymers [97, 98]. Most recently, antifreeze proteins have been tested to maintain mist quality of fire hydrants in subzero environments [99].

Ice nucleation proteins, mainly from *Pseudomonas syringae*, have been used in the recreation and food industries. These environmentally safe and efficient proteins have replaced inorganic equivalents such as silver iodide [96]. Able to induce ice crystal formation at warmer temperatures, ice nucleation proteins have also been used to create artificial snow (e.g., Snowmax) for recreational use. For food applications, ice nucleation proteins have been used in juice and beer preparation. These proteins induce freezing at higher temperature allowing a more energy efficient concentrating process [100].

Outside of the industry, ice nucleation proteins have research applications as well. Exploiting the cell surface anchoring capabilities, recombinant proteins linked to ice nucleation proteins have been expressed as fusion proteins to be displayed on the cell surface of the expression host [101]. Only limited by proper folding, these heterologous proteins have been used as biosensors and biosorbents. More importantly, this system can eventually lead to the cell surface display of antigenic epitopes. Consequently, a live bacterium expressing a recombinant antigen, fused to anchored ice nucleation proteins, on its cell surface can be used as a vaccine delivery system to elicit an immune response. Another research application includes using ice nucleation proteins as a reporter gene. One study used the protein to measure the transcriptional activity of *ipdC* and *iaaM* genes [102]. Transcription levels were measured based on ice nucleation activity of the fusion where activity increases as the square of protein abundance [103].

Instead of purified proteins, viable ice nucleation active bacteria are being considered for agricultural use as biocontrol agents for insect pests. Transgenic *Enterobacter cloacae *expressing an *ina* gene from *Erwinia ananas* was shown to increase the freezing temperature of corn borer and cotton bollworm following ingestion [61]. Tang and colleagues [62] report stable colonization in insect larvae by the transgenic bacterium for at least 9 days and poor colonization in plants. This method was seen to be quite effective, as 80% of treated larvae froze after exposure to −5°C for 3 hours and 100% of treated larvae froze at −7°C for 12 hours. However, one major drawback of this approach is the lack of immediate results. Thus, farmers are likely to opt for different methods of pesticide control.

Both ice crystal controlling proteins are prevalent globally in a wide variety of psychrophilic bacteria. With a focus on freeze tolerance, bacterial antifreeze proteins are known for a low level of non-colligative freezing point depression with greater emphasis on ice recrystallization inhibition. Superior ice recrystallization inhibition is utilized for improved survivability against freeze-thaw stress common to temperate, non-Antarctic environments. On the other hand, bacterial ice nucleation proteins induce ice crystal formation at high subzero temperatures and can influence precipitation in the atmosphere. For both antifreeze and ice nucleation proteins, only a small number of bacterial sources of these enzymes have been studied in any detail and very few of these bacterial enzymes have been isolated and characterized at the protein structure level. However, the suggested mechanism for both protein classes features the same ice crystal interactions, using an ordered ice-like hydration layer, with protein functions differentiated based on protein size.

## Figures and Tables

**Table 1:** Documented ice nucleation active bacteria.

Bacterial genus and species	Reference
*Erwinia ananas *	[1, 104]
*Erwinia herbicola *	[1, 48, 105]
*Erwinia uredovora *	[1, 49]
*Erwinia carotovora*	[1, 50]
*Pseudomonas antarctica *	[51, 106]
*Pseudomonas aeruginosa *	[107]
*Pseudomonas borealis *	[13, 15]
*Pseudomonas fluorescens *	[1, 14, 108]
*Pseudomonas putida *	[4, 7, 11, 33]
*Pseudomonas syringae *	[1, 31, 42, 46, 104, 109]
*Pseudomonas viridiflava *	[106]
*Pantoea ananatis *	[110]
*Pantoea agglomerans *	[105]
*Xanthomonas campestris *	[69, 111]

Bacterial genus	

*Bacillus, Coliwellia, Clavibacter, Corynebacterium, Curtobacterium, Exiguobacterium, Flavobacterium, Frigoribacterium, Kluyvera, Pedobacter, Pseudoxanthomonas, Sphingobacterium, *and *Sphingomonas *	[6, 41, 60, 69, 112]
